# Nanopore sequencing in suitcase lab enables improved detection of *β*-lactamase genes in food-borne *E. coli*

**DOI:** 10.3389/fmicb.2026.1854040

**Published:** 2026-07-16

**Authors:** Prakash Ghosh, Ulrike Binsker, Rea Maja Kobialka, Arianna Ceruti, Muhammad Asaduzzaman, Siddhartha Narayan Joardar, Asaduzzaman Asad, Behrouz Alizadeh Savareh, Michael Frimpong, Bhupesh Taneja, Martin Siegel, Yakhya Dieye, Uwe Truyen, Ahmed Abd El Wahed

**Affiliations:** 1 Institute for Animal Hygiene and Veterinary Public Health, Leipzig University, Leipzig, Germany; 2 Department of Empirical Health Economics, Technische Universität Berlin, Berlin, Germany; 3 Nutrition Research Division, International Centre for Diarrhoeal Disease Research, Bangladesh (icddr,b), Dhaka, Bangladesh; 4 One Network for Environment and Bioscience (ONEB), Dhaka, Bangladesh; 5 Department Biological Safety, German Federal Institute for Risk Assessment, Berlin, Germany; 6 Department of Community Medicine and Global Health, Institute of Health and Society, Faculty of Medicine, University of Oslo, Oslo, Norway; 7 Department of Veterinary Microbiology, West Bengal University of Animal and Fishery Sciences, Kolkata, India; 8 Gut-Brain Axis Laboratory, Infectious Diseases Division, ICDDR, B, Dhaka, Bangladesh; 9 Department of Molecular Medicine, School of Medicine and Dentistry, Kwame Nkrumah University of Science and Technology, Kumasi, Ghana; 10 CSIR-Institute of Genomics and Integrative Biology (CSIR-IGIB), New Delhi, India; 11 Chair of General Economics, Health Economics and Econometrics, University of Greifswald, Greifswald, Germany; 12 Pole de Microbiologie, Institut de Pasteur de Dakar, Dakar, Senegal

**Keywords:** antimicrobial resistance, *E. coli*—*Escherichia coli*, nanopore sequencing, suitcase lab, *β*-lactamase genes

## Abstract

**Background:**

The application of next-generation sequencing (NGS) is rapidly expanding for antimicrobial resistance (AMR) surveillance and clinical decision-making. However, despite a high AMR burden, many low- and middle-income countries lack the infrastructure and technical capacity required to implement sequencing-based approaches. To address this gap and demonstrate the practical application of the technology, we compared the performance of nanopore sequencing combined with real-time analysis using the EPI2ME platform in a mobile laboratory with that of a hybrid sequencing approach integrating Illumina short reads and nanopore long reads.

**Methods:**

In this exploratory diagnostic evaluation study, 25 *Escherichia coli* isolates obtained through the German annual national AMR monitoring program were included. Isolates resistant to at least one *β*-lactam antibiotic were considered as resistant isolate. Genomic DNA was subjected to both Illumina and nanopore sequencing. Following the sequencing, the EPI2ME whole genome sequencing pipeline was used to detect and identify *β*-lactamase genes in *E. coli* isolates. As the comparator, both long and short read-based hybrid assemblies were used to detect and identify the *β*-lactamase genes. The sensitivity, specificity, positive predictive value (PPV), and negative predictive value (NPV) of the sequencing approaches were calculated against the phenotypic classification of *E. coli* isolates, treating the presence or absence of *β*-lactamase–encoding genes as the genotypic predictor of *β*-lactam resistance.

**Results:**

A total of 36 *β*-lactamase–encoding genes, predominantly from the *bla*_CTX-M_, *bla*_SHV_, and *bla*_TEM_ families, were detected. Both analytical approaches achieved a promising accuracy towards predicting *β*-lactam resistance. Moderate agreement was observed between methods, with an overall concordance of 68.9%. A distinct distribution pattern of *β*-lactamase genes was observed across the MIC ranges of the isolates for third-generation cephalosporins and the carbapenems.

**Discussion:**

These findings support the feasibility of deploying nanopore sequencing in mobile suitcase laboratories to strengthen AMR surveillance in food production systems. Given its portability, rapid turnaround time, and minimal infrastructure requirements, the approach may also have broader applications for One Health based AMR surveillance, clinical decision-making, and outbreak investigations in resource-limited settings.

## Introduction

Antimicrobial resistance (AMR), a major public health problem, is associated with 1.27 million deaths every year, along with substantial morbidity (Murray et al., 2022). With significant mortality and morbidity, AMR might incur a cumulative economic cost of 100 trillion USD per year by 2050(Nelson et al., 2021). Besides, it is assumed that AMR might result in a death toll of about 10 million people each year in the coming decades (O’Neill, 2016). A high-resolution observation in the global burden of AMR reveals that this silent pandemic is disproportionately affecting the developing world (Murray et al., 2022). Eventually, discerning the importance of ensuring antimicrobial stewardship (AMS) and enhanced monitoring of the AMR transmission, the World Health Organization (WHO) initiated the Global Antimicrobial Resistance and Use Surveillance System (GLASS) in 2015. For particularly AMR surveillance, GLASS defines *Escherichia coli* as the sentinel organism because of its ubiquity across human, animal, food, and environmental interfaces. According to the GLASS report of 2020, the observed rate of *E. coli* resistance to 3rd generation cephalosporins in low and middle-income countries (LMICs) was three times higher than that of high-income countries (HICs), whereas the HICs reported 10 times more AST data as compared to that of LMICs (GLASS Report: Early Implementation, 2020). The aforementioned paradox clearly signifies the suboptimal coverage and quality of AMR surveillance data in the developing countries. Like the AMR surveillance, antimicrobial stewardship programs (ASPs) are also poorly implemented in many developing countries, limiting efforts to de-escalate inappropriate antibiotic use (Tiong et al., 2016). Particularly, *β*-lactam antibiotics are the most frequently used antimicrobials across the globe (Narendrakumar et al., 2022). However, due to the widespread and irrational use of *β*-lactam antibiotics in human medicine, animal and agriculture sectors, resistance to this superlative drug class has emerged in most of the clinically important bacterial pathogens (Narendrakumar et al., 2022). Consequently, *β*-lactamase genes, the major determinants of *β*-lactam resistance, are frequently harboured on mobile genetic elements and they play a critical role in the transmission of resistance across human, animal, and environmental interfaces (Bento et al., 2025). Therefore, innovative methods and strategies are highly appreciated to track the *β*-lactam-resistant markers in bacterial isolates for both surveillance and clinical decision-making.

To date, antimicrobial susceptibility testing (AST) has been considered the gold standard for investigating the resistance phenotypes of bacterial isolates against certain classes of antibiotics, including *β*-lactam antibiotics (Pereckaite et al., 2018). Especially in the low-resource settings, AMR surveillance mostly relies on the AST (Global Antimicrobial Resistance and Use Surveillance System -- GLASS-AMR Manual 2.0, n.d.). However, owing to the long turnaround time and operational complexity of culture-based approaches, efforts are ongoing to develop molecular approaches. In line with such approaches, Nucleic acid amplification tests (NAATs) have been utilized for AMR detection, including *β*-lactam resistant markers, but these methods are developed for limited targets and are resource demanding (Vasala et al., 2020). To overcome the limitations of conventional target-based molecular methods, whole-genome sequencing (WGS) has become an alternative approach that demonstrates strong agreement between genotypic resistance determinants and phenotypic resistance profiles in bacterial isolates. A previous study showed that WGS reliably predicts phenotypic resistance, particularly for *β*-lactams and ESBL-producing *E. coli* (Doyle et al., 2020).

Due to the numerous advantages of genomic surveillance, a wide range of sequencing technologies and platforms are being applied in tracking of AMR genes and plasmids, with each technology offering distinct benefits and limitations (Thorpe et al., 2024). Recently, the long-read sequencing technique developed by Oxford Nanopore Technology (ONT) has become popular for real-time pathogen surveillance. Unlike short-read sequencing, ONT is capable of identifying complex genomic features like repeat elements, structural variants, and plasmids that leverage the accurate bacterial species identification and their corresponding AMR determinants (Weinmaier et al., 2023; Foster-Nyarko et al., 2023). Owing to the portability, speed, and scalability, this rapid sequencing technique has been employed for pathogen surveillance, outbreak response, and AMR monitoring in both field and clinical settings (Struelens et al., 2024; Ferreira et al., 2021). Notably, Thorpe et al. showed enhanced performance of ONT towards tracking of multidrug-resistant tuberculosis from whole genomes and assembling plasmids compared to Illumina sequencing (Thorpe et al., 2024). Here to add, previously we applied nanopore sequencing for the detection of bacteria in wastewater as well (Schurig et al., 2024). Despite the advantages of NGS platforms, sequence analysis still remains a challenge in deploying this technique in remote settings. Especially, the command-based approaches for detection and identification of the ARGs require advanced skills and knowledge in bioinformatics. According to Ras et al. research institutions and healthcare facilities in LMICs encounter an array of challenges including the shortage of infrastructure, lack of training facilities, poor internet, and lack of local expertise towards implementation of the sequencing-based techniques (Ras et al., 2021). To address these constraints and democratize access to a sequencing-based approach, ONT has introduced a bacterial WGS pipeline in EPI2ME for accurate and early detection of AMR and acquiring a high-resolution picture of the bacterial genome, which is yet to be evaluated and validated. However, in addition to challenges in sequence analysis pipelines, scaling up sequencing in low-resource settings remains difficult due to limited access to laboratories equipped with the necessary sequencing infrastructure.

With this backdrop, to facilitate the detection of AMR in low-resource settings, we incorporated the nanopore sequencing into a mobile suitcase laboratory, which has been deployed in different countries for on-site detection of infectious pathogens and managing outbreaks (Kobialka et al., 2025). However, the evidence for the accuracy and feasibility of mobile suitcase laboratory-based nanopore sequencing integrated with the EPI2ME WGS pipeline for AMR detection is very limited. To address this gap and establish a practical use case, we conducted this study to evaluate the performance of nanopore sequencing coupled with analysis through EPI2ME in a mobile suitcase laboratory and compared it with a hybrid sequencing approach combining Illumina and nanopore platforms. Using 25 *E. coli* isolates, we assessed the ability of each approach to predict *β*-lactam resistance phenotypes based on the presence or absence of *β*-lactamase genes.

## Method and materials

### Study design and selection of the isolates

Our current study was an exploratory diagnostic evaluation designed to assess the efficiency of nanopore sequencing in a suitcase lab for rapid detection of AMR determinants associated with *β*-lactamase resistance. In this study, total 25 *E. coli* isolates were included which were obtained in the framework of the annual national monitoring program for Germany in accordance with Directive 2003/99/EC and Commission implementing decisions (CID) 2013/652/EU and 2020/1729/EU. The *E. coli* used in this study were isolated from the meat of chickens, cattle, and pigs as well as caecal and faecal samples from broilers, turkeys, ducks, calves, breeding sows, and fattening pigs by regional laboratories and send to the National Reference Laboratory for Antimicrobial Resistance (NRL-AR) hosted at BfR for species confirmation (i.e., growth on indicator media), matrix-assisted laser desorption ionization–time of flight [MALDI-ToF] and antimicrobial susceptibility testing (AST) (Pauly et al., 2021). The sources of the isolates under investigation are listed in Supplementary material 1.

**Figure 1:**
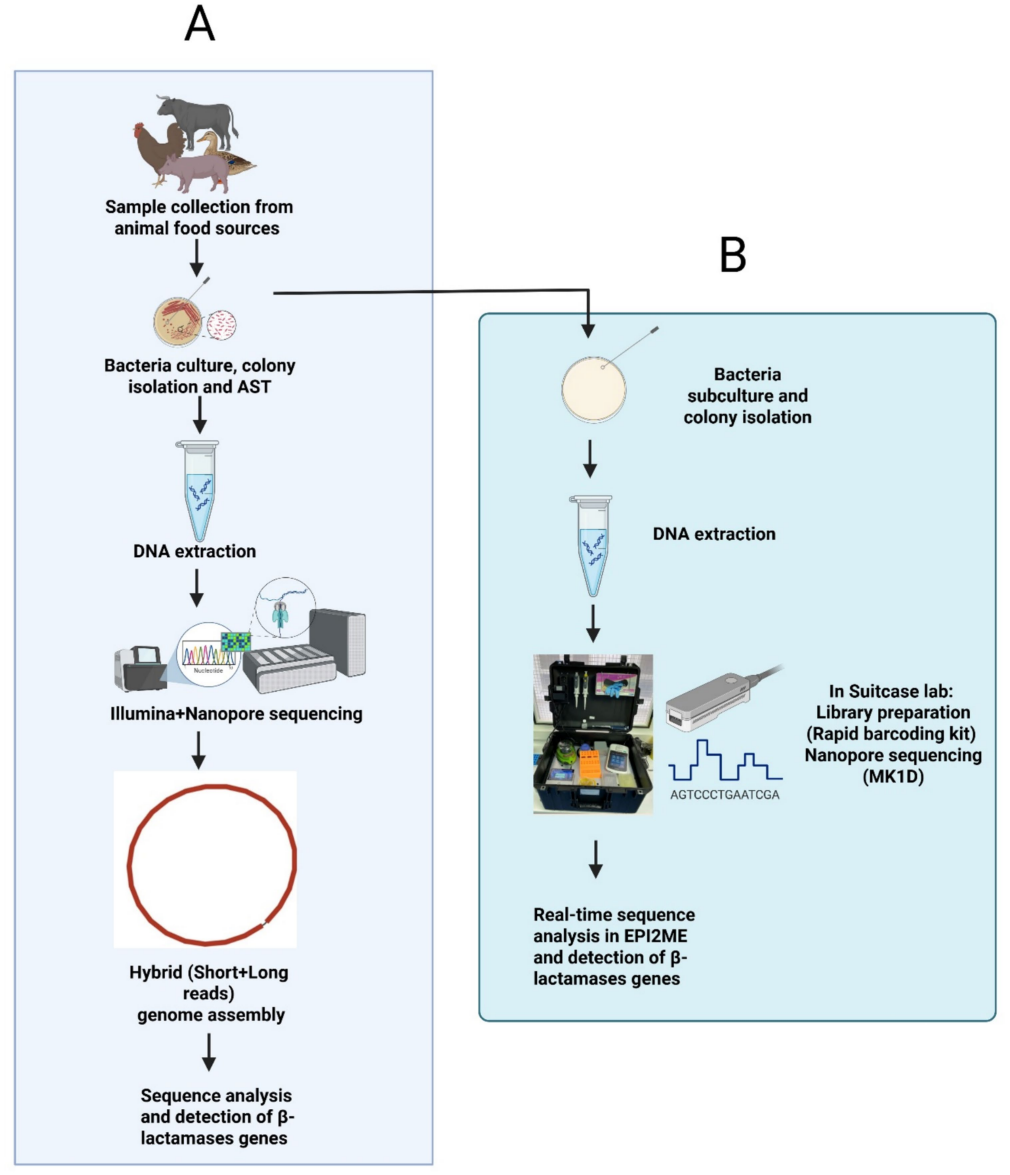
Comparative workflows for the detection of *β*-lactamase genes in *E. coli* isolates using conventional hybrid whole-genome sequencing and a nanopore sequencing approach in mobile suitcase laboratory. **(A)** Reference workflow performed at the German Federal Institute for Risk Assessment (BfR). *E. coli* isolates obtained from animal-derived food sources underwent bacterial culture, colony isolation, antimicrobial susceptibility testing (AST), and genomic DNA extraction. DNA was sequenced using both Illumina short-read and Oxford Nanopore long-read platforms. The sequencing data were combined through hybrid assembly to generate high-quality near-complete bacterial genomes (represented by the circular genome), which were subsequently analysed for the detection and characterization of *β*-lactamase genes. **(B)** Workflow performed in the mobile suitcase laboratory at Leipzig University (ULEI). Following bacterial subculture, colony isolation, and DNA extraction, sequencing libraries were prepared using the Rapid Barcoding Kit and sequenced on a portable MinION Mk1D device. Nanopore reads were analysed in real time using the EPI2ME Bacterial Whole Genome Sequencing workflow (ONT-EPI2ME), enabling automated genome assembly, polishing, and rapid detection of *β*-lactamase genes. Created with BioRender.com.

Among the isolates, 24 were resistant to at least one *β*-lactam antibiotic and one isolate served as a negative control. In line with the objective of the study, we did perform next generation sequencing (NGS) on 25 *E. coli* isolates. Both Illumina and Nanopore sequencing were performed at the German Federal Institute for Risk Assessment (BfR) and bioinformatic analysis was performed with the hybrid assembly to detect and identify the *β*-lactamase genes. With the same isolates, nanopore sequencing was performed in the suitcase lab at Leipzig University (ULEI). Following the sequencing, the EPI2ME whole genome sequencing pipeline was used to detect and identify *β*-lactamase genes in *E. coli* isolates (Figure 1).

### Antimicrobial susceptibility testing (AST)

AST of *E. coli* isolates was conducted by broth microdilution according to the guidelines of the Clinical and Laboratory Standards Institute (CLSI) M07 (12th ed.) as described previously (Binsker et al., 2023). In brief, a commercial Sensititre system (Thermo Scientific, Meerbusch, Germany) with a harmonized European panel of 15 antimicrobial substances of 12 antimicrobial classes (EUVSEC3) and 10 antimicrobial substances (EUVSEC2) was used in concentration ranges described in Commission Implementing Decision (CID) 2020/1729/EU. Epidemiological cut-off values (ECOFFs) were used for evaluation according to the guidelines of the European Committee on Antimicrobial Susceptibility Testing (EUCAST) and the CID 2020/1729/EU. Isolates that showed a specific pattern, as defined by EFSA using the EUVSEC2 plate, were referred to as extended-spectrum *β*-lactamase (ESBL)-/AmpC *β*-lactamase (AmpC)−/carbapenemase (CP)-producing *E. coli* (European Food Safety Authority and European Centre for Disease Prevention and Control, 2025) The AST findings for the *β*-lactam antibiotics are included in the Supplementary material 2.

### DNA extraction, whole-genome sequencing and bioinformatic analyses at BfR

Genomic DNA was extracted from cell pellets of 1 mL overnight cultures grown in LB using the PureLink™ Genomic DNA Mini Kit (Invitrogen, Thermo Fisher Scientific). Sequencing libraries were prepared using the DNA Prep (M) Tagmentation Kit (former name Nextera DNA Flex; Illumina, USA) utilising paired-end reads and sequenced on a MiSeq device (2 × 301 bp cycles) or on a NextSeq 500 device (2 × 151 or 2 × 149 bp cycles, Illumina Inc.) (Lienen et al., 2021). The quality assessment of raw reads, trimming, *de novo* assembly and quality assessment, taxonomic identification and contamination checks were performed using the in-house AQUAMIS pipeline^1^ (Deneke et al., 2021). The BakCharak pipeline (version 3.0.4),^2^ which runs AMRFinder, was used to analyze the assembled short-read data for their antimicrobial resistance genes. Long-read sequencing libraries were prepared using a Rapid Barcoding Kit 96 (V11 or V14; Oxford Nanopore Technologies [ONT], UK) and sequenced on either an ONT MinION™ Mk1C device, a MinIT v19.12.5, or a P2 Solo using an R9.4.1 or 10.4.1 MinION Flow Cell. Base calling that converts sequencing data from signal (.fast5) to sequence format (.fastq) was performed using Guppy and the “fast base calling” option.

The MiLongA pipeline^3^ was used to analyze the raw long reads (Frentzel et al., 2025). This included read trimming and quality filtering, as well as generating hybrid assemblies of the short and long reads using the Unicycler v0.4.4 assembler. Based on this assembly, resistance genes were confirmed using ResFinder version 4.7.2 provided by the Danish Technical University (http://www.genomicepidemiology.org, accessed 23rd June 2025).

### DNA extraction at ULEI

DNAs were extracted using the Qiagen DNeasy Blood and Tissue kit (QIAGEN, Hilden, Germany), according to the manufacturer’s recommendations. Briefly, 5–10 pure isolated colonies were picked and homogenized into the lysis buffer, followed by DNA purification and collection into sterile tubes. A sample with sterile water was used as a blank control to monitor background DNA. The quality and concentration of extracted DNA were evaluated using Qubit 3.0 fluorometer (ThermoFisher Scientific) with the 1× dsDNA HS Assay kit (Invitrogen, Q33231) and adjusted when needed for library preparation.

### Library preparation, sequencing and read processing in the suitcase lab

The mobile laboratory consisted of a portable suitcase (56 × 45.5 × 26.5 cm) containing all essential equipment and consumables required for DNA quality assessment, library preparation, and nanopore sequencing. The setup was powered by a solar panel and portable power pack, enabling up to 18 h of continuous operation under field conditions. In the mobile suitcase lab, the MinION sequencing libraries with DNA samples were prepared with the Rapid Barcoding Sequencing Kit (SQK-RBK114.96) with the extracted DNA. Briefly, 10 μL of 200 ng of template DNA was mixed with 1.5 μL of Fragmentation Mix RB01-25, and the tube was incubated at 30 °C for 2 min and then at 80 °C for 2 min. All the barcoded samples were pooled, and 1 μL of Rapid Adapter (RAP) was added to 11 μL of barcoded DNA, followed by incubation for 5 min at room temperature. The sequencing was performed in a portable MinION sequencing device with MinION R10.4.1 flow cells. Sequencing was started with MinKNOW™ software (V25.05.14), and was used to perform base-calling (High Accuracy) and barcode demultiplexing during sequencing and through post-run analysis.

### Bioinformatic analysis and resistance genes detection using EPI2ME

Following the previous step in mobile suitcase lab, sequence analysis was performed using a standard laptop connected to the internet, allowing real-time processing of sequencing data through cloud-based bioinformatic workflows. As per the workflow, the generated FASTQ-pass reads were sent through the EPI2ME workflow to align the sequences and identify antibiotic resistance genes in real time. The bioinformatic analysis was carried out in EPI2ME Bacterial Whole Genome workflow (V1.4.3), which includes quality control and statistics of the filtered and trimmed reads by fastcat/Bamstats, *De novo* genome assembly by Flye, assembly polishing by medaka and resistance gene identification using the ResFinder (V4.7.2). The resistance genes being identified by the workflow’s protein homolog model were retained only, which is the most conservative model, and excluded resistance genes with detection accuracy below 90% according to the protein homolog model to minimize the false positive rate.

### Statistical analysis

The sensitivity, specificity, positive predictive value (PPV), and negative predictive value (NPV) of the sequencing approaches were calculated against the phenotypic classification of *E. coli* isolates, treating the presence or absence of *β*-lactamase–encoding genes as the genotypic predictor of *β*-lactam resistance. Agreement between the approaches was determined using Cohen kappa (*κ*). To explore the relationship between antimicrobial resistance determinant acquisition and phenotypic resistance, *β*-lactamase genes detected by both approaches were mapped across the minimum inhibitory concentration (MIC) ranges of third-generation cephalosporins (Cefotaxime, Cefoxitin, and Ceftazidime) and the carbapenems (Meropenem, Imipenem, Ertapenem). Besides, the relation or association between sequencing depth and the number of detected beta-lactamase genes was determined through Pearson correlation. Statistical significance was assigned at a *p* value of ≤0.05. All analyses were performed in R (V4.5.1) and Python (V3.13.5).

## Results

### Distribution and characterization of the *β*-lactamase genes

**Figure 2:**
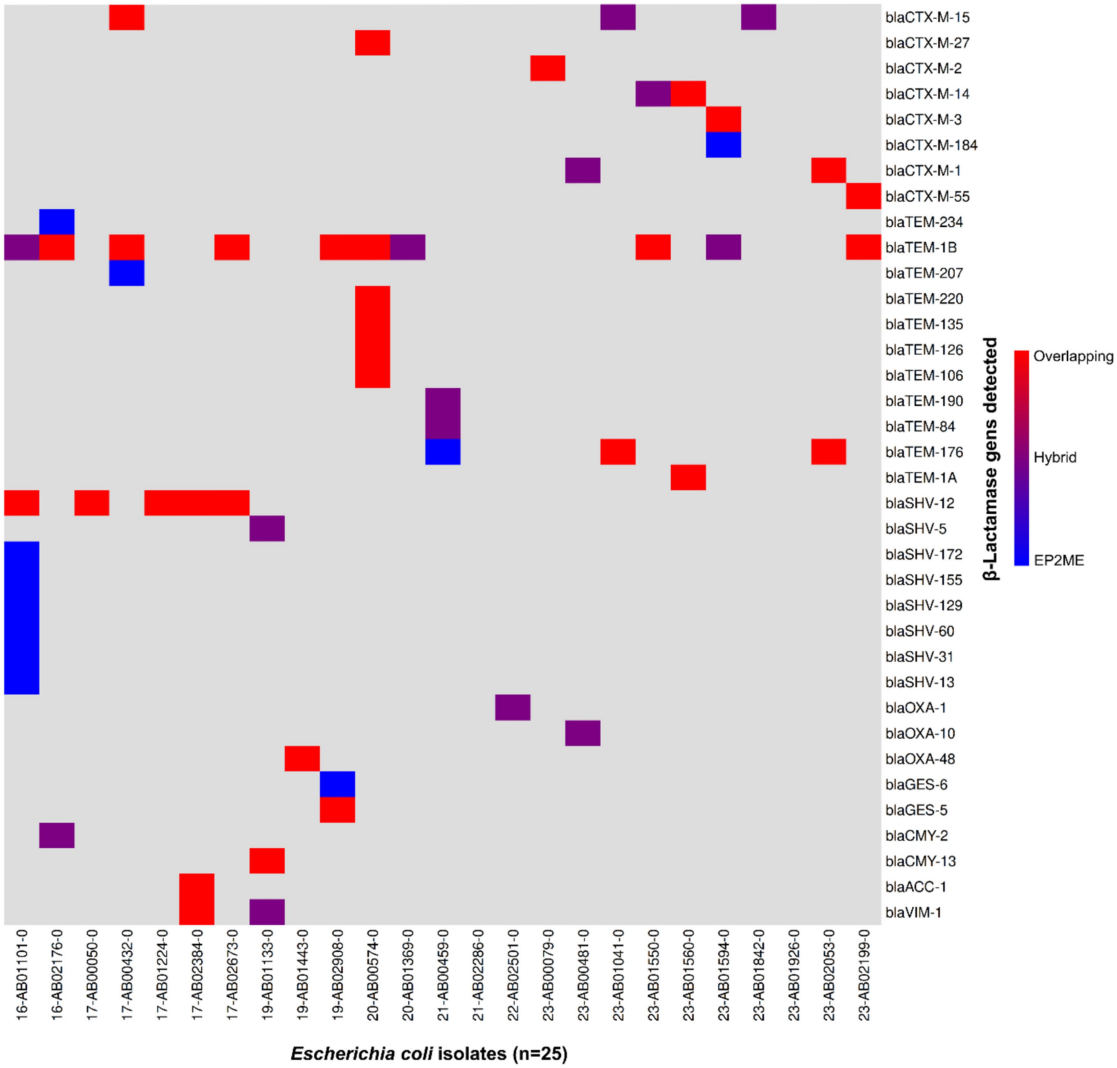
Hierarchical clustering of *β*-lactamase genes across *E. coli* isolates. Created with BioRender.com.

A total of 36 *β*-lactamase–encoding markers belonging to multiple gene classes were detected using the EPI2ME and hybrid assembly approaches. Both methods commonly identified 21 resistance genes, while 10 genes were detected exclusively by EPI2ME and five genes exclusively by the hybrid assembly approach (Supplementary material 3). Most of the *β*-lactamase genes belonged to the CTX-M, SHV and TEM classes. Identified variants included *bla*_CTX-M_^1,2,3,14,15,27,55,184^,*bla*_SHV_^5,12,13,31,60,129,155,172^ and *bla*_TEM_^1A,1B,84,106,126,135,176,190,207,220,234^ (Figure 2). In addition, genes with beta-lactam hydrolysing capacity, including *bla*_GES_^5,6^, *bla*_CMY_^2,13^, *bla*_OXA_^1,10,48^, *bla*_VIM_^1^ and *bla*_ACC_^1^ were detected.

**Figure 3:**
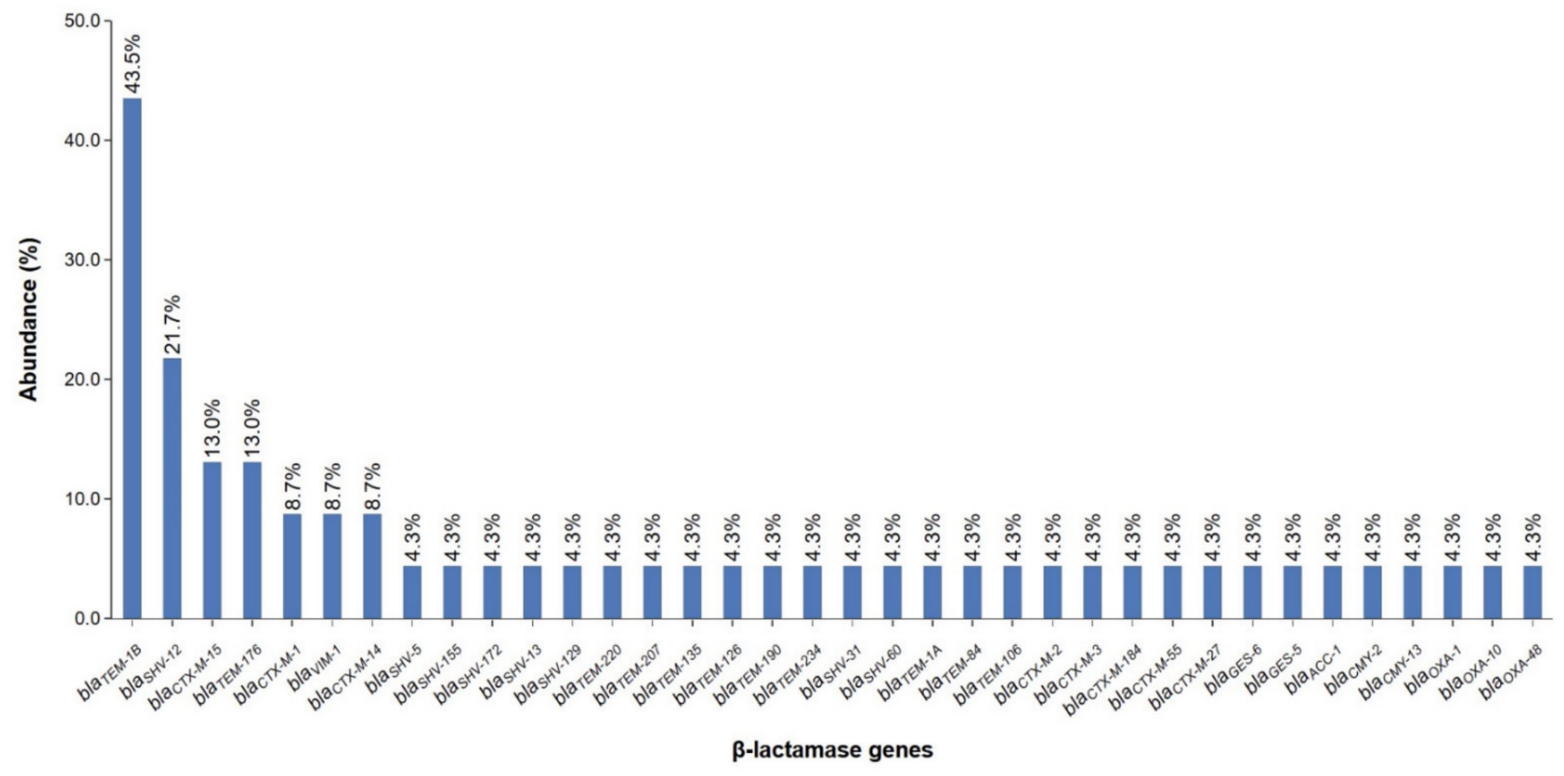
Distribution of all *β*-lactamase genes being detected through ONT-EPI2ME and/or hybrid approaches among the *E. coli* isolates. Created with BioRender.com.

The distribution of *β*-lactam antibiotic resistance genes varied across the *E. coli* isolates. We found that about 43% of the isolates contained *bla*_TEM-1B_, 22% isolates *bla*_SHV-12_, 13% isolates *bla*_TEM-176_ and 13% isolates *bla*_CTX-M-15_ (Figure 3). Besides, 8.7% of the isolates were detected with *bla*_CTX-M-14_, *bla*_CTX-M-1_ and *bla*_VIM-1_. A more granular analysis revealed that among the 24 ß-lactam resistant isolates, 21 isolates were detected with a ARGs from the CTX-M, SHV and TEM classes by both or any of the analytical approaches. To be specific, 58% of the resistant isolates were detected with at least one variant from the CTX-M class followed by 45% with TEM class where 25% of the resistant isolates were detected with genes from SHV class. The remaining genes were detected at least once by both or one analytical approaches. Notable to mention, *bla*_GES-5_, *bla*_ACC-1_, *bla*_VIM-1_, *bla*_CMY-13_ and *bla*_OXA-48_ were detected by both methods. Interestingly, one resistant isolate, 23-AB01926-0 was not detected with any *β*-lactam antibiotic resistance genes. Following analysis of the whole genome sequencing data of the isolate through the BakCharak pipeline, a single nucleotide variant was detected and identified on the promoter region of the AmpC gene coding sequence.

### Performance of NGS in predicting the resistance phenotype

**Figure 4:**
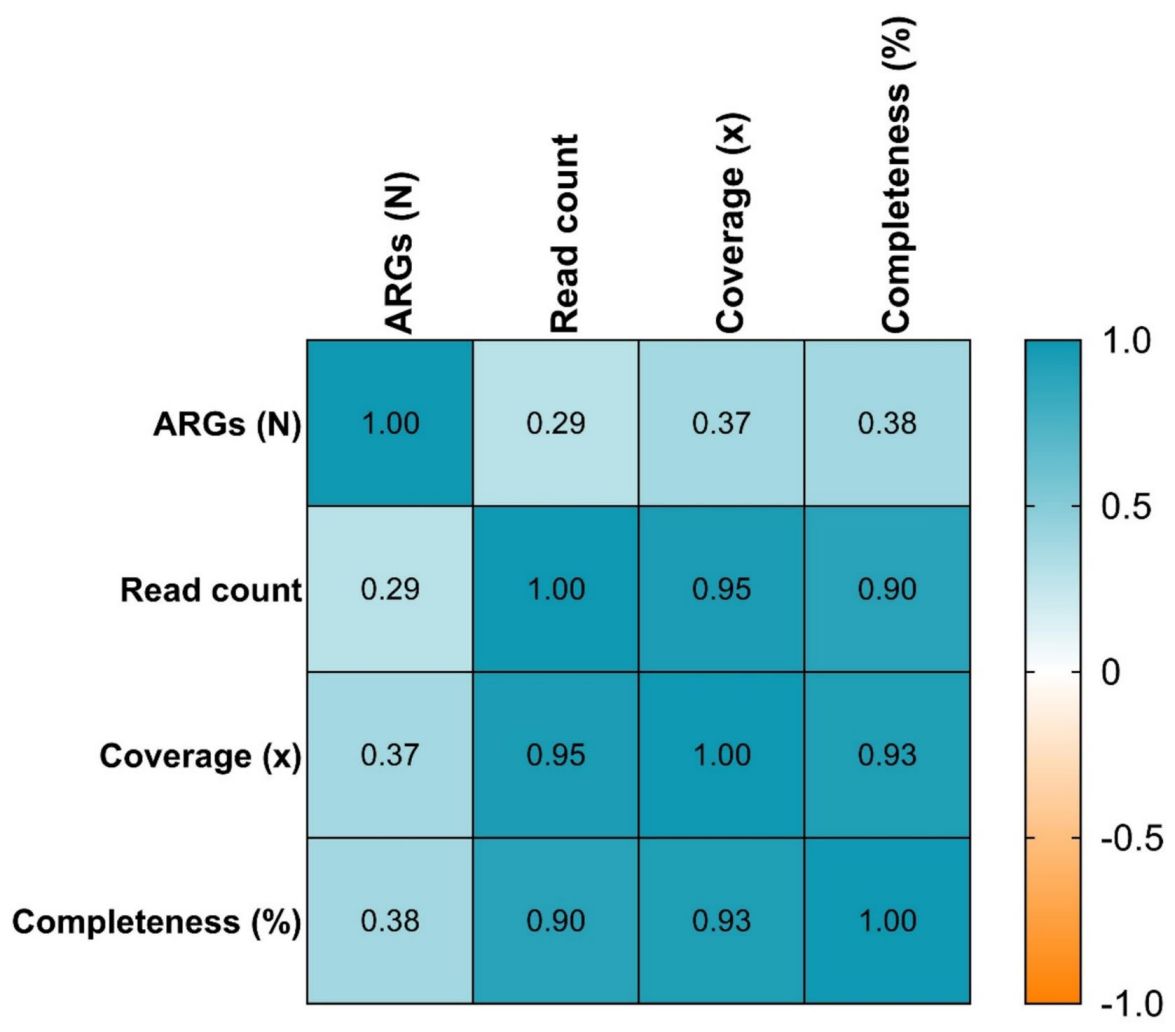
Correlation between the sequencing depth (genome coverage, read counts, genome completeness) and the number of detected *β*-lactamase genes for ONT-EPI2ME approach. Created with BioRender.com.

Since one of the phenotypically resistant isolates lacked the *β*-lactamase gene and one isolate was susceptible to *β*-lactam antibiotics, 23 isolates were considered cases and two isolates were considered controls to assess the efficacy of the analytical approaches. Both analytical approaches demonstrated excellent sensitivity for the detection of *β*-lactamase genes (Table 1). Likewise, absolute specificity was observed for both of the methods. Besides, both of the approaches demonstrated an absolute positive predictive value (PPV). In contrary, EPI2ME showed a comparatively lower negative predictive value (NPV) than that of the hybrid approach. Overall, promising accuracy (>80%) was observed for EPI2ME whereas absolute accuracy (100%) was observed for the hybrid approach. The two methods showed moderate agreement, with an overall concordance of 68.88% in detecting *β*-lactamase genes. Besides, we found a weak correlation between sequencing depth and the number of detected *β*-lactamase genes. Through Pearson correlation test, we found a poor relation of the average read counts, genome coverage and genome completeness with the number of *β*-lactamase genes being detected (Figure 4).

**Table 1:** The efficiency of ONT-EPI2ME and hybrid methods in predicting the *β*-lactam resistant phenotype.

	ONT-EPI2ME	Hybrid
Sensitivity	82.61% (61.22–95.05%)	100% (85.18–100%)
Specificity	100.00% (15.81–100%)	100% (15.81–100%)
PPV	100% (82.35–100%)	100% (85.18–100%)
NPV	33.33% (17.02–54.92%)	100% (15.81–100%)
Accuracy	84.00% (63.92–95.46%)	100% (86.28–100%)
Kappa	*κ* = 0.43 or Moderate agreement	

### Relation between acquisition of AMR determinants and MICs of *β*-lactam antibiotics

**Figure 5:**
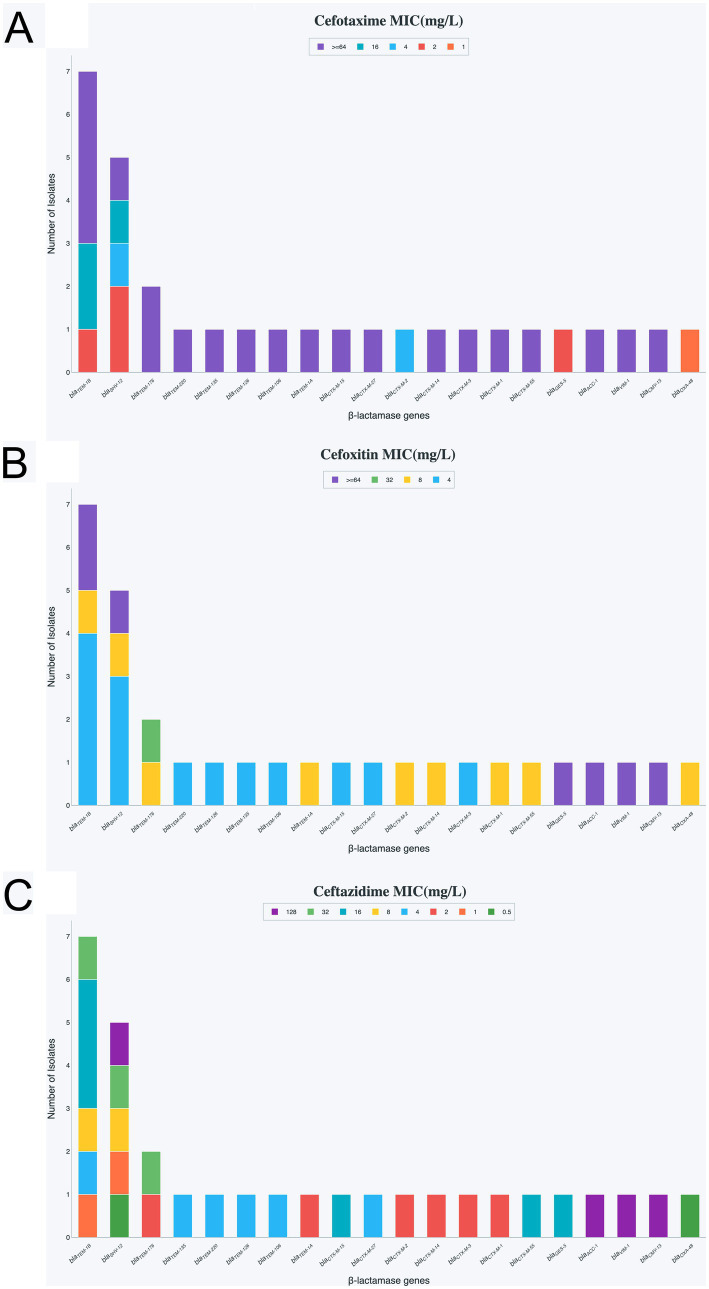
Distribution of *β*-lactamase genes in isolates with MICs being determined through AST (EUVSEC2/3) against third generation cephalosporins (Cefotaxime, Cefoxitin, and Ceftazidime). Created with BioRender.com.

**Figure 6:**
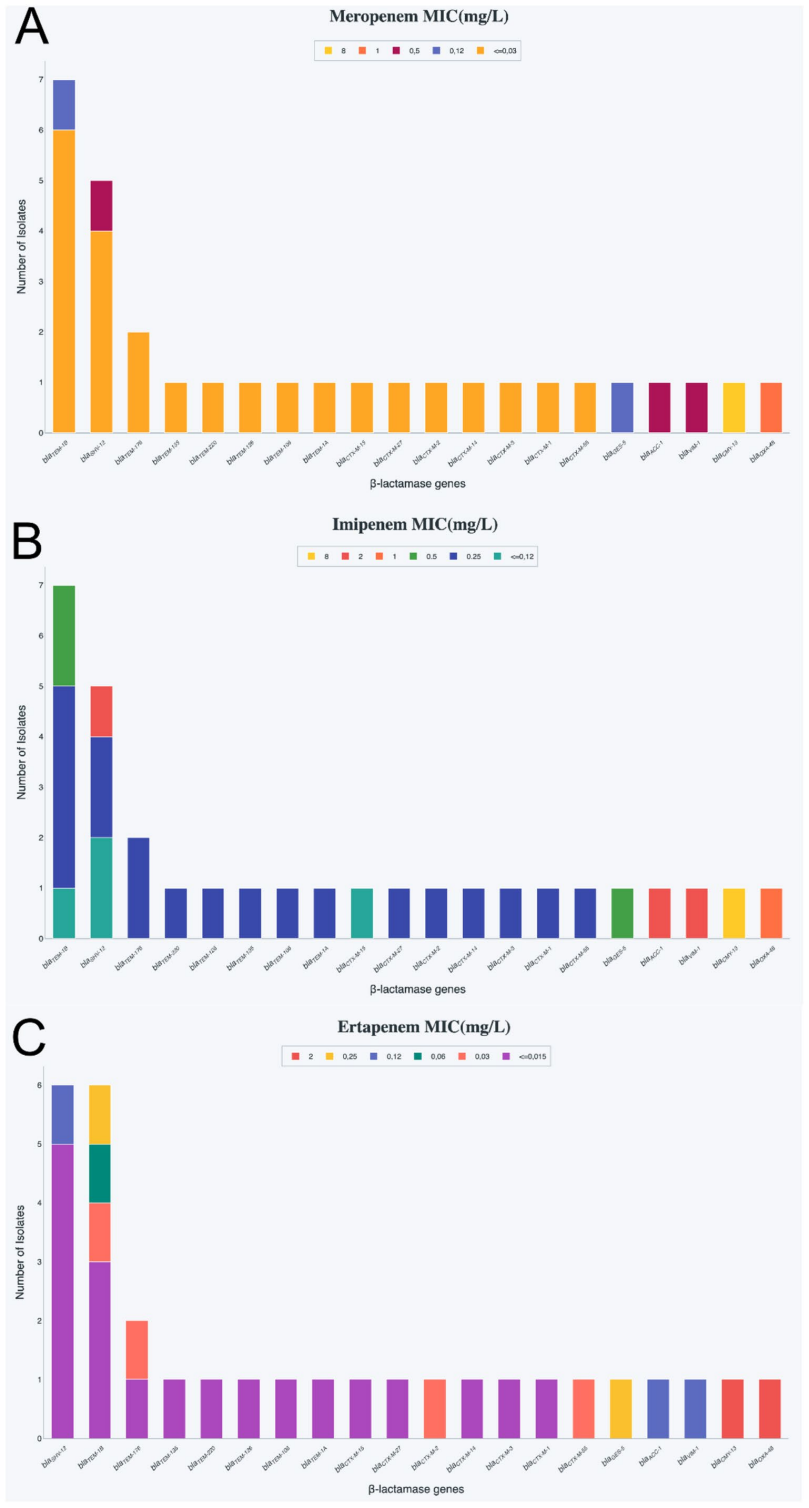
Distribution of *β*-lactamase genes in isolates with MICs being determined through AST (EUVSEC2/3) against carbapenems (Meropenem, Imipenem, Ertapenem). Created with BioRender.com.

For Ampicillin, all of the resistant isolates showed an MIC >32 mg/L, whereas we observed variable MIC index against the third-generation cephalosporins (Cefotaxime, Cefoxitin, and Ceftazidime) (Figure 5) and the carbapenems (Meropenem, Imipenem and Ertapenem) among the isolates harbouring *β*-lactamase genes (Figure 6). For cefotaxime, the majority of *β*-lactamase genes were detected in isolates with the highest MIC level (64 mg/L), while a commonly found ESBL gene, *bla*_SHV-12_ was observed in isolates with MICs ranging from 1 to 64 mg/L. In the case of cefoxitin, *bla*_TEM_ and *bla*_SHV-12_ were detected in isolates with MICs ranging from 4 to 64 mg/L, with most *bla*_CTX-M_, *bla*_SHV_, and *bla*_TEM_ variants clustering in isolates exhibiting MICs between 4 and 8 mg/L. For ceftazidime, *β*-lactamase genes were present in isolates with diverse MIC levels, although the majority of *bla*_CTX-M_, *bla*_SHV_, and *bla*_TEM_ genes were identified in isolates with MICs between 2 and 4 mg/L. Regarding meropenems, genes from *bla*_CTX-M_*, bla*_TEM_ and *bla*_SHV_ classes were detected and identified across the entire concentration ranges of the antibiotic. Notably, isolates harbouring AmpC *β*-lactamase (*bla*_ACC_ and *bla*_CMY_) and carbapenemase (*bla*_GES_*, bla*_VIM_*, and bla*_OXA_) genes consistently exhibited the highest MIC values.

## Discussion

With the increased burden of mortality and morbidity related to AMR infections, it is imperative to improve the speed and accuracy of bacterial AMR detection and identification (Shelburne et al., 2017). Apart from clinical decision-making, AMR surveillance has raised concerns about tackling this silent pandemic. The transmission of AMR determinants follows an intricate pathway comprising One Health domains: human, animal, and environment interfaces. Eventually, the detection and identification of the AMR markers from different sources and the triangulation of data are important to explore their emergence, escalation, and transmission (Babu Rajendran et al., 2023). Considering the existing gaps and unmet needs, an accurate and rapid sequencing-based tool is a prerequisite to facilitate AMR surveillance both in high-income and low-income settings. Therefore, the overarching goal of this study was to evaluate the accuracy of nanopore sequencing coupled with real-time analysis of sequencing data in EPI2ME, which requires minimal bioinformatic knowledge. To implement the method in low-resource settings or at point-of-need, the sequencing was performed in a portable modular platform, a mobile suitcase lab.

To the best of our knowledge, this is the first study evaluating the performance of nanopore sequencing deployed in a mobile suitcase laboratory and coupled with the EPI2ME analysis (Bacterial Whole Genome Sequencing workflow) for real-time detection of *β*-lactamase genes in *E. coli*. The investigative approaches showed promising agreement where both of the sequencing approaches demonstrated high accuracy in predicting the *β*-lactamase resistance phenotype from the targeted *β*-lactamase genes. A recent study reported promising efficiency of nanopore sequencing towards the detection of ARGs conferring the third-generation cephalosporin resistance in enterobacteria with a sensitivity and specificity of 81 and 56.3%, respectively (Kawang et al., 2024). Besides, Shelburne S et al. demonstrated 87% sensitivity and 98% specificity of Illumina sequencing in predicting the *β*-lactam resistant phenotype of gram-negative bacteria from the resistance conferring *β*-lactamase genes (Shelburne et al., 2017). The findings of the previous studies along with the promising results of the current study, corroborate the application of NGS for AMR surveillance and clinical decision making (Ogunleye et al., 2025). In the current study, the enhanced efficiency of nanopore sequencing in predicting the phenotype can be attributed to several factors. Firstly, the advancement in the flow cell chemistry (R10.4.1) and base calling algorithm has made the nanopore sequencing data more accurate (Q12–15). Besides, the new bacterial whole genome sequence analysis pipeline uses medaka polisher which increases the quality of the long read based genome assemblies (Foster-Nyarko et al., 2023). Finally, in the current study, the antibiotic and AMR determinants combination was narrowed down to *β*-lactam class whereas in our previous study, we considered 12 antibiotic classes (Ogunleye et al., 2025).

Being the AMR determinants against the *β*-lactam antibiotics, genes belonging to *bla*_CTX-M_, *bla*_TEM_ and *bla*_SHV_ classes were found to be abundant in the current study, where all of the *E. coli* isolates were obtained from the food chain. A previous study demonstrated that the genes from CTX-M class are the most abundant ESBL conferring AMR determinants for *E. coli* in livestock (Kaesbohrer et al., 2019; Di Marcantonio et al., 2025). A recent meta-analysis performed in Bangladesh found *bla*_CTX-M_ in 87.8%, *bla*_TEM_ in 71.7% and *bla*_SHV_ in 17.9% of ESBL-positive enterobacterial, demonstrating the dominance of CTX-M in animals (Rahaman et al., 2025). Recently, we reported multiple clinically relevant alleles, including *bla*_TEM-164_, *bla*_CTX − M-15_, and *bla*_SHV-45_ in *Salmonella* isolates of India obtained from duck, underscoring their potential public health significance (Paul et al., 2026). Recently, another review reported the prominence of ARGs from these three classes globally in animal sources (Tseng et al., 2023). The aforementioned evidences substantiate distribution of ARGs in *E. coli* isolates in the current study. Among the detected ARGs with extended-spectrum cephalosporins hydrolysing capacity, *bla*_SHV-12_ was found to be prevalent. This SHV variant is one of the most widespread ESBL genes detected in *E. coli* and *Salmonella* from food-producing animals, particularly in poultry (Di Marcantonio et al., 2025). In recent times, SHV-12 has been increasingly reported in poultry farms, farm environments, meat products, and in some cases in livestock workers, indicating its ability to spread across the One Health interface (Dahms et al., 2015; Saliu et al., 2017). Its dissemination is primarily associated with IncF, IncI1, and IncX plasmids, which enable efficient horizontal transmission between bacteria in animal production systems (Carattoli, 2009; Jie et al., 2021).

Apart from the highly transmissible genes of CTX, SHV and TEM classes, few other *β*-lactamase gene classes, including clinically-relevant carbapenemases, (*bla*_GES_*, bla*_ACC_*, bla*_VIM_*, bla*_CMY_ *and bla*_OXA_) were identified as well. Genes from these classes are associated with hospital outbreaks and severe infection, however, these are less abundant as their carriers (plasmids, integrons) are less transmissible (Fadare and Okoh, 2021). On the other hand, genes from CTX-M, SHV and TEM classes spread through IncF, IncI, IncN, and IncA/C plasmids which are host-adapted to *E. coli*, highly stable, and easily transferable (Fadare and Okoh, 2021). Among the 24 resistant isolates, one isolate was not detected with any *β*-lactamase gene due to a mutation in the promoter region of the *ampC* gene, resulting in phenotypic resistance. This instance substantiated the specificity of EPI2ME in the detection of ARGs. As expected, no *β*-lactamase gene was detected in the susceptible isolate.

In addition to the detection and identification of the *β*-lactamase genes, another important investigation of this study was to explore the relation between the acquisition of specific *β*-lactamase genes and phenotypic resistance as defined by the MICs against the *β*-lactam antibiotics. In most cases, the genes from *bla*_CTX-M_, *bla*_SHV_ and *bla*_TEM_ classes were detected in the isolates with comparatively low MICs for cephalosporins and carbapenems, whereas AmpC *β*-lactamase and carbapenemase genes were detected in the isolates with comparatively high MICs. Such scenario may be explained by the co-existence of *β*-lactamase and carbapenemase genes that likely result in additive or synergistic hydrolytic activity, thereby substantially increasing MICs and limiting the effectiveness of *β*-lactam antibiotics (Poirel et al., 2004). A recent study indicated that ARGs and resistance mutations were associated with an increased MIC where a subset of ARGs were found to be putatively independently confer resistance (Lipworth et al., 2025). In addition, that study found differential effects of acquired ARGs between different generations of cephalosporins. The findings of our study also align with the previous study.

### Limitations of the study

Notwithstanding the promising findings, the current study encountered some methodological limitations. Firstly, the number of samples in this study was limited which might have negatively impacted the diagnostic accuracy of the investigative approaches. However, the objective of the study was to make a use case as well as to validate the new approaches. Secondly, the depth of the nanopore sequencing data in the suitcase lab was not up to the mark as expected which might have impacted the detection and identification of the ARGs. Previous studies demonstrate that ≥30× whole-genome coverage is required for accurate detection of AMR genes and reliable AMR prediction (Wu et al., 2023; Thornval et al., 2025). The low depth of the sequencing data can be attributed to the low concentration of the DNA samples (<1 ng/ul) being used in the current study. Consequently, low-input samples may yield fewer reads, shorter read lengths, and incomplete genome coverage, potentially reducing the sensitivity of AMR gene detection and genomic assembly quality (Wick et al., 2019; Tyler et al., 2018). Therefore, the previous studies emphasized the use of DNA samples with higher concentration (Kawang et al., 2024; Thornval et al., 2025). Such finding substantiates the use of the most effective DNA extraction method and extraction kit to get high DNA yield while ensuring purity. Previous studies showed how the extraction methods influenced the downstream assembly performance and the detection of AMR determinants in resistant bacterial isolates (Bari et al., 2025; Thornval et al., 2025; Purushothaman et al., 2024).

In the current study, a validated commercial DNA extraction kit was employed, however, the DNA yield obtained from several isolates was lower than anticipated. The DNA extraction output is influenced not only by the extraction chemistry itself but also by factors such as bacterial biomass, isolate-specific characteristics, storage conditions, and the practical constraints associated with field-deployable laboratory workflows. Previous studies have demonstrated considerable variability in DNA quantity and quality among bacterial isolates despite the use of standardized extraction protocols (Bari et al., 2025; Thornval et al., 2025; Purushothaman et al., 2024). As the primary objective of this study was to evaluate the feasibility of a mobile suitcase laboratory under real-world conditions, additional laboratory-based optimization procedures were not performed. Consequently, several samples yielded DNA concentrations below those generally recommended for optimal Oxford Nanopore sequencing, which may have resulted in reduced sequencing depth, incomplete genome coverage, and lower sensitivity for antimicrobial resistance gene detection.

Besides, we did not affirm the presence of the resistance genes through PCR. However, the use of hybrid assembly as the comparator complemented the limitation as well as improved the accuracy of the alternative approach.

### Key implications and future directions

To date, phenotypic antimicrobial susceptibility testing has been regarded as the gold standard for both surveillance and clinical decision-making (Hattab et al., 2024). However, with the increasing concordance between genotypic and phenotypic resistance profiles, the integration of sequencing-based approaches has been strongly advocated by experts, including the Lancet Commission on AMR (Jauneikaite et al., 2023). In this context, nanopore sequencing deployed through a mobile suitcase laboratory, coupled with user-friendly bioinformatic pipelines, represents a significant advance towards implementing sequencing technologies in low-resource settings. Moreover, this approach has been found simple and less resource demanding compared to the existing long reads and short reads-based hybrid assembly approach while ensuring optimum accuracy in predicting the *β*-lactam resistant phenotypes of the *E. coli* isolates. Notably, despite low sequence depth, EPI2ME correctly identified most of the clinically important *β*-lactamase genes where the specificity of the approach is commendable. Eventually, the proposed approach will pave the way to strengthen OH-AMR surveillance along with AMS in resource-poor settings. It is worth mentioning that nanopore sequencing has advanced considerably compared with other sequencing platforms, particularly in terms of turnaround time and per-sample cost. A recent study demonstrated that nanopore sequencing can be cost-competitive, with estimated per-sample costs ranging from approximately USD 25, excluding capital equipment and downstream bioinformatic analysis (Kawang et al., 2024). Moreover, its operational complexity is substantially lower than that of alternative sequencing approaches. Notably, the use of the V14 rapid barcoding kit reduces library preparation time to approximately 1 h, whereas other sequencing workflows typically require several hours to days (Zidane et al., 2025).

To further reduce turnaround time, efforts are ongoing to develop culture-independent sequencing workflows. Recent evidence has shown excellent concordance between the abundance of AMR determinants detected directly from raw clinical samples and from cultured isolates using metagenome-assembled genomes (MAGs) generated from nanopore sequencing data (Campos-Madueno et al., 2024). The incorporation of MAG-based analytical pipelines into platforms such as EPI2ME could therefore enable direct detection and characterization of AMR genes from clinical and environmental samples at the point of need. In parallel, the adoption of rapid and simplified DNA extraction methods may further streamline the transition to point-of-need detection of AMR (Madaje et al., 2025). Besides, advances in sequencing automation suggest that increasingly cost-effective and field-deployable solutions will soon become integral components of diagnostic and surveillance systems in resource-constrained settings (Siddall et al., 2025).

## Conclusion

The present study demonstrates high-accuracy detection of *β*-lactamase genes, which is critical for both clinical decision making and surveillance, particularly for understanding the dynamics of AMR across human, animal, and environmental interfaces. In addition, the detection of mobile genetic element (MGE)associated genes facilitates genomic epidemiological investigations, thereby strengthening OH-AMR surveillance. Overall, the findings together with the evidences from previous study provide a strong rationale for the deployment of nanopore sequencing in mobile suitcase laboratories for OH-AMR surveillance, clinical decision-making, and outbreak investigations in low-resource settings (Ogunleye et al., 2025). The applicability of long-read whole-genome sequencing for AMR surveillance in resource-constrained settings is further corroborated by a recent study from Sri Lanka, which successfully implemented an ONT-based sequencing workflow for the detection and characterization of antimicrobial resistance determinants (Kumari et al., 2025). This approach is particularly well-suited for laboratories with access to culture facilities but limited or no sequencing infrastructure, and it may serve as an effective makeshift solution during outbreak investigations in healthcare settings. Nevertheless, further methodological optimization and validation will be required to support broader implementation and long-term scalability. In particular, inclusion of diverse antibiogram combinations is essential to support both surveillance and antimicrobial stewardship objectives. To make suitcase laboratories fully point-of-need deployable, the aforementioned advancements are currently being pursued under the ongoing initiatives of the ADAPT One Health Network to strengthen AMR surveillance and antimicrobial stewardship capacity in sub-Saharan Africa (Abd El Wahed et al., 2024).

## Data Availability

The original contributions presented in the study are publicly available. This data can be found here: https://www.ncbi.nlm.nih.gov/sra, accession number PRJNA1428713.

## Footnotes

1
https://gitlab.com/bfr_bioinformatics/AQUAMIS/

2
https://gitlab.com/bfr_bioinformatics/bakcharak

3
https://gitlab.com/bfr_bioinformatics/milonga
