# Supplementary material for: Nanopore sequencing in suitcase lab enables improved detection of β-lactamase genes in food-borne E. coli
Source: Front Microbiol. 2026 Jul 16;17:1854040. doi: 10.3389/fmicb.2026.1854040 (PMC13420423; doi:10.3389/fmicb.2026.1854040)
Supplement: Supplementary file 1 [file Table_1.docx]

| **Isolate ID** | **Source** | **Illumina Device** | **Library Prep Kit** | **ONT device** | **Library Prep Kit** | **Barcoding Kit** | **Flow cell** | **Data availability** |
| --- | --- | --- | --- | --- | --- | --- | --- | --- |
| 16-AB01101 | broiler, caecum | MiSeq | Nextera DNA Flex | Mk1C | SQK-RBK110.96 | RBK11096.10.0009 | R9.4.1 | PRJNA721573 |
| 16-AB02176 | broiler, faeces | NextSeq | Nextera DNA Flex | Mk1C | SQK-RBK110.96 | RBK11096.10.0009 | R9.4.1 | PRJNA1306484 |
| 17-AB00050 | broiler, caecum | NextSeq | Nextera DNA Flex | MinIT v19.12.5 | SQK-RBK110.96 | RBK11096.10.0009 | R9.4.1 | PRJNA589028 |
| 17-AB00432 | Veal, caecum | MiSeq | Nextera DNA Flex | Mk1C | SQK-RBK110.96 | RBK11096.10.0009 |  | PRJNA589028 |
| 17-AB01224 | Fattening pig, faeces | MiSeq | Nextera DNA Flex | Mk1C | SQK-RBK110.96 | RBK11096.10.0009 | R9.4.1 | PRJNA721573 |
| 17-AB02384 | Fattening pig, caecum | MiSeq | Nextera DNA Flex | P2 Solo | SQK-RBK114.96 | RBK1496.30.0002 | R10.4.1 | PRJNA721573 |
| 17-AB02673 | Fattening pig, faeces | NextSeq | Nextera DNA Flex | P2 Solo | SQK-RBK114.96 | RBK1496.30.0002 | R10.4.1 | PRJNA721573 |
| 19-AB01133 | Pork meat | MiSeq | Nextera DNA Flex | Mk1C | SQK-RBK110.96 | RBK11096.10.0009 | R9.4.1 | MT682138 |
| 19-AB01443 | Fattening pig, faeces | MiSeq | Nextera DNA Flex | Mk1C | SQK-RBK110.96 | RBK11096.10.0009 | R9.4.1 | WOWW00000000 and MT193824 |
| 19-AB02908 | Fattening pig, faeces | NextSeq | Nextera DNA Flex | Mk1C | SQK-RBK110.96 | RBK11096.10.0009 | R9.4.1 | PRJNA660949 and  MT955355 |
| 20-AB00574 | Turkey, caecum | NextSeq | Nextera DNA Flex | Mk1C | SQK-RBK110.96 | RBK11096.10.0009 | R9.4.1 | PRJNA726012 |
| 20-AB01369 | Chicken, meat | NextSeq | DNA Prep (M) Tagmentation | Mk1C | SQK-RBK110.96 | RBK11096.10.0009 | R9.4.1 | PRJNA1306484 |
| 21-AB00459 | Veal, faeces | NextSeq | DNA Prep (M) Tagmentation | Mk1C | SQK-RBK110.96 | RBK11096.10.0009 | R9.4.1 | PRJNA1306484 |
| 21-AB02286-0 | Cattle meat | NextSeq | DNA Prep (M) Tagmentation | Mk1C | SQK-RBK110.96 | RBK11096.10.0004 | R9.4.1 | PRJNA1306484 |
| 22-AB02501-0 | Duck,  faeces | NextSeq | DNA Prep (M) Tagmentation | Mk1C | SQK-RBK110.96 | RBK11096.10.0004 | R9.4.1 | PRJNA1306484 |
| 23-AB00079-0 | Chicken, meat | NextSeq | DNA Prep (M) Tagmentation | P2 Solo | SQK-RBK114.96 | RBK1496.30.0002, | R10.4.1 | PRJNA1306484 |
| 23-AB00481-0 | Breeding sow, faeces | NextSeq | DNA Prep (M) Tagmentation | P2 Solo | SQK-RBK114.96 | RBK1496.30.0002, | R10.4.1 | PRJNA1306484 |
| 23-AB01041-0 | Veal, caecum | NextSeq | DNA Prep (M) Tagmentation | P2 Solo | SQK-RBK114.96 | RBK1496.30.0002, | R10.4.1 | PRJNA1306484 |
| 23-AB01550-0 | Veal, caecum | NextSeq | DNA Prep (M) Tagmentation | P2 Solo | SQK-RBK114.96 | RBK1496.30.0002, | R10.4.1 | PRJNA1306484 |
| 23-AB01560-0 | Breeding sow, faeces | NextSeq | DNA Prep (M) Tagmentation | P2 Solo | SQK-RBK114.96 | RBK1496.30.0002, | R10.4.1 | PRJNA1306484 |
| 23-AB01594-0 | Fattening pig, caecum | NextSeq | DNA Prep (M) Tagmentation | P2 Solo | SQK-RBK114.96 | RBK1496.30.0002, | R10.4.1 | PRJNA1306484 |
| 23-AB01842-0 | Veal, caecum | NextSeq | DNA Prep (M) Tagmentation | P2 Solo | SQK-RBK114.96 | RBK1496.30.0002, | R10.4.1 | PRJNA1306484 |
| 23-AB01926-0 | Fattening pig, caecum | NextSeq | DNA Prep (M) Tagmentation | P2 Solo | SQK-RBK114.96 | RBK1496.30.0002, | R10.4.1 | PRJNA1306484 |
| 23-AB02053-0 | Veal, caecum | NextSeq | DNA Prep (M) Tagmentation | P2 Solo | SQK-RBK114.96 | RBK1496.30.0002, | R10.4.1 | PRJNA1306484 |
| 23-AB02199-0 | Wild duck, caecum | NextSeq | DNA Prep (M) Tagmentation | P2 Solo | SQK-RBK114.96 | RBK1496.30.0002, | R10.4.1 | PRJNA1306484 |

| **Isolate ID** | **Sequencing serial** | **ESBL (R=resistant, S=susceptible)** | **Coverage**  **(Long reads)** | **Coverage**  **(Short reads)** | **Genome completeness** |
| --- | --- | --- | --- | --- | --- |
| 16-AB01101-0 | 01 | R | 48.22X | 109.97X | 99.97 |
| 16-AB02176-0 | 02 | R | 48.03X | 187.01X | 99.33 |
| 17-AB00050-0 | 03 | R | 141.22X | 153.83X | 99.55 |
| 17-AB00432-0 | 04 | R | 147.38X | 183.69X | 99.40 |
| 17-AB01224-0 | 05 | R | 81.32X | 92.67X | 99.97 |
| 17-AB02384-0 | 06 | R | 137.64X | 44.29X | 99.93 |
| 17-AB02673-0 | 07 | R | 60.41X | 70.36X | 99.33 |
| 19-AB01133-0 | 08 | R | 34.29X | 31.69X | 99.02 |
| 19-AB01443-0 | 09 | R | 274.30X | 138.22X | 99.98 |
| 19-AB02908-0 | 10 | R | 79.52X | 110.60X | 99.70 |
| 20-AB00574-0 | 11 | R | 33.03X | 62.08X | 99.20 |
| 20-AB01369-0 | 12 | S | 141.14X | 80.91X | 99.43 |
| 21-AB00459-0 | 13 | S | 58.50X | 62.79X | 99.15 |
| 21-AB02286-0 | 14 | S | 166.07X | 77.16X | 99.58 |
| 22-AB02501-0 | 15 | S | 194.32X | 67.84X | 99.63 |
| 23-AB00079-0 | 16 | R | 234.59X | 61.96X | 99.85 |
| 23-AB00481-0 | 17 | R | 166.79X | 60.09X | 99.69 |
| 23-AB01041-0 | 18 | R | 142.13X | 75.16X | 99.55 |
| 23-AB01550-0 | 19 | R | 123.87X | 57.34X | 99.37 |
| 23-AB01560-0 | 20 | R | 199.46X | 55.04X | 99.23 |
| 23-AB01594-0 | 21 | R | 116.78X | 48.85X | 99.28 |
| 23-AB01842-0 | 22 | R | 185.23X | 76.12X | 99.70 |
| 23-AB01926-0 | 23 | R | 113.41X | 72.63X | 99.34 |
| 23-AB02053-0 | 24 | R | 128.96X | 54.17X | 99.38 |
| 23-AB02199-0 | 25 | R | 163.60X | 54.50X | 99.60 |
