# Supplementary material for: Nanopore sequencing in suitcase lab enables improved detection of β-lactamase genes in food-borne E. coli
Source: Front Microbiol. 2026 Jul 16;17:1854040. doi: 10.3389/fmicb.2026.1854040 (PMC13420423; doi:10.3389/fmicb.2026.1854040)
Supplement: Supplementary file 2 [file Table_2.DOCX]

Table: The AST findings of the 25 E. coli isolates against the β-lactam antibiotics
