# Supplementary material for: Nanopore sequencing in suitcase lab enables improved detection of β-lactamase genes in food-borne E. coli
Source: Front Microbiol. 2026 Jul 16;17:1854040. doi: 10.3389/fmicb.2026.1854040 (PMC13420423; doi:10.3389/fmicb.2026.1854040)
Supplement: Supplementary file 3 [file Table_3.DOCX]

| ONT-EPI2ME approach | Hybrid assembly approach | Detected by both approaches |
| --- | --- | --- |
| **blaSHV-172**  **blaSHV-155**  **blaSHV-129**  **blaSHV-60**  **blaSHV-31**  **blaSHV-13**  **blaTEM-234**  **blaTEM-207**  **blaCTX-M-184**  **blaGES-6** | **blaSHV-5**  **blaTEM-190**  **blaTEM-84**  **blaCMY-2**  **blaOXA-10** | **blaSHV-12**  **blaTEM-1B**  **blaTEM-220**  **blaTEM-135**  **blaTEM-126**  **blaTEM-106**  **blaTEM-176**  **blaTEM-1A**  **blaCTX-M-15**  **blaCTX-M-27**  **blaCTX-M-2**  **blaCTX-M-14**  **blaCTX-M-3**  **blaCTX-M-1**  **blaCTX-M-55**  **blaGES-5**  **blaACC-1**  **blaVIM-1**  **blaCMY-13**  **blaOXA-1**  **blaOXA-48** |

Table: Distribution of the AMR genes detected by two different approaches
